# Automated Assessment of Breast Positioning Quality in Screening Mammography

**DOI:** 10.3390/cancers14194704

**Published:** 2022-09-27

**Authors:** Mouna Brahim, Kai Westerkamp, Louisa Hempel, Reiner Lehmann, Dirk Hempel, Patrick Philipp

**Affiliations:** 1 Fraunhofer Institute of Optronics, System Technologies and Image Exploitation IOSB, Fraunhofer Center for Machine Learning, 76131 Karlsruhe, Germany; 2 Medical School, Sigmund Freud University, 1090 Vienna, Austria; 3 DontBePatient Intelligence GmbH, 20095 Hamburg, Germany; 4 Institute of Translational Molecular Tumor Research, 85354 Freising, Germany

**Keywords:** decision support, mammogram, positioning quality, breast cancer, cranio-codal, mediolateral oblique, deep learning, convolutional neuronal networks, trustworthy AI

## Abstract

**Simple Summary:**

Inadequate breast positioning quality is the main cause behind misdiagnosis of breast cancer in screening mammography. For this reason, the first step before any cancer diagnosis is to ensure that the acquired mammograms have adequate breast positioning quality according to predefined criteria. If this is not the case, the patient must return for a new mammography. In this study, we proposed an approach for an automatic assessment of breast positioning quality in screening mammography using Convolutional Neural Networks. Eventually, this approach is not intended to replace radiology technicians, but rather to assist them in identifying inadequately positioned mammograms in real time, reduce the number of returned patient and improve the efficiency of cancer detection. For each predefined criterion, a specific convolutional neural network was separately trained and then combined into an overall system that predicts whether the breast is well positioned or not, achieving an efficient accuracy of 96.5% for craniocaudal and 93.3% for mediolateral oblique images. Our approach differs from already available studies and commercial tools by taking into account more useful breast positioning criteria that have to be considered by the expert, and thus providing a more holistic assistance.

**Abstract:**

Screening mammography is a widely used approach for early breast cancer detection, effectively increasing the survival rate of affected patients. According to the Food and Drug Administration’s Mammography Quality Standards Act and Program statistics, approximately 39 million mammography procedures are performed in the United States each year. Therefore, breast cancer screening is among the most common radiological tasks. Interpretation of screening mammograms by a specialist radiologist includes primarily the review of breast positioning quality, which is a key factor affecting the sensitivity of mammography and thus the diagnostic performance. Each mammogram with inadequate positioning may lead to a missed cancer or, in case of false positive signal interpretation, to follow-up activities, increased emotional burden and potential over-therapy and must be repeated, requiring the return of the patient. In this study, we have developed deep convolutional neuronal networks to differentiate mammograms with inadequate breast positioning from the adequate ones. The aim of the proposed automated positioning quality evaluation is to assist radiology technologists in detecting poorly positioned mammograms during patient visits, improve mammography performance, and decrease the recall rate. The implemented models have achieved 96.5% accuracy in cranio-caudal view classification and 93.3% accuracy in mediolateral oblique view regarding breast positioning quality. In addition to these results, we developed a software module that allows the study to be applied in practice by presenting the implemented model predictions and informing the technologist about the missing quality criteria.

## 1. Introduction and Related Work

Breast cancer is one of the most common tumors in women worldwide. According to current statistics published by the World Health Organization, 2.3 million women worldwide were diagnosed with breast cancer in 2020 and around 685,000 died from it [1]. Screening mammography is the primary imaging exam used to detect breast cancer at its early stages and has been shown to reduce the mortality rate by approximately 30% [2]. It includes two standard views, cranial-caudal (CC) and mediolateral-oblique (MLO) views of each breast, resulting in a set of four images. To achieve precise diagnostics and increase the patient´s survival rate, high-quality mammographic images are required [3,4].

The U.S. Food and Drug Administration (FDA) has issued a statement citing poor mammography positioning as the main cause of most clinical image deficiencies and most misdiagnoses [5]. Therefore, each mammogram must first be reviewed by a specialized radiologist for proper positioning before a final diagnosis is made. Once the predefined criteria regarding breast positioning quality are not met, the patient will be recalled for additional imaging. Repeating a mammography examination is inconvenient for both medical staff and the patient. It results in additional radiation exposure for the patient as well as anxiety from delayed results and increased workload for the technologist and radiologist. Moreover, it leads to extra costs and time consumed unnecessarily.

To minimize this issues, deep learning methods may be applied to automatically recognize inadequately positioned mammograms and provide immediate feedback to the mammography technologist. This way, we can reduce the number of returning patients with initially improper image quality and improve the efficiency of cancer detection on screening mammography by ensuring the visualization of all breast tissue.

With the introduction of full-field digital mammography systems in the last two decades and the recently enormous success of deep learning in object recognition and image classification, automation of medical image interpretation has become a subject of intense interest. Despite the various approaches in medicine [6,7,8], many recent studies have demonstrated the potential of applying convolutional neural networks (CNNs) in medical imaging—including mammography. However, most of them have exclusively focused on the classification of breast cancer abnormalities [1,9,10,11]. The assessment of positioning quality has so far received relatively little attention in medical image analysis and none of the available studies have achieved high performance in this area, despite its importance for effective breast cancer detection. In the last year, an interesting research paper has been published, presenting a deep learning algorithm for automatic detection of poorly positioned mammograms [12]. In this approach, not all standard defined quality criteria related to breast positioning were evaluated. Only the height of the pectoralis muscle in mediolateral oblique view and the presence of retroglandular fat in cranio-caudal view were considered. In addition, the developed algorithm was trained and tested with a small number of mammograms for both views. This was primarily due to the lack of availability of large public labeled datasets.

## 2. Materials and Methods

The first required task in this study was obtaining a sufficient number of mammograms to build effective image classification models. Collecting medical images is a challenge in itself. Publicly available datasets were created for research-based applications and studies for automated breast cancer detection. Two of the most well-known and widely used public datasets are DDSM [13] and MIAS [14], with respectively 2620 and 322 scanned film mammography studies. The digitization of these mammograms aggravated the problem of pre-existing noise and low contrast issues, making the automated evaluation of breast positioning according to predefined quality criteria difficult and sometimes near-impossible.

**Figure 1:**
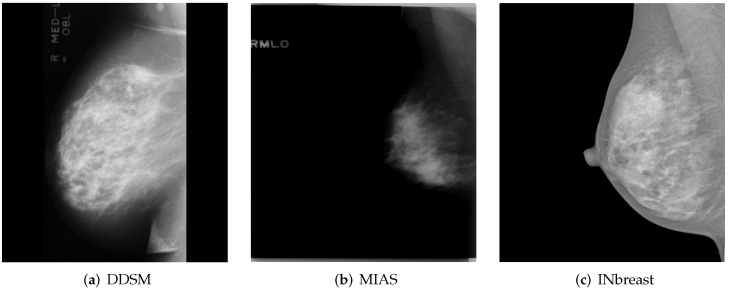
MLO views of the right breast from publicly available datasets. (**a**,**b**) A sample from the DDSM [13] and MIAS [14] dataset, respectively. (**c**) A sample from INbreast [15]. Reprinted/adapted with permissions from [13,14,15].

INbreast [15] is another public dataset that contains a total of 115 full-field digital mammography studies with CC and MLO views for each breast. These mammograms have different intensity profiles and higher image resolution compared to digitized film mammograms from the DDSM and MIAS datasets. Figure 1 shows a sample (MLO view of the right breast) from each of the previously mentioned publicly available datasets.

**Figure 2:**
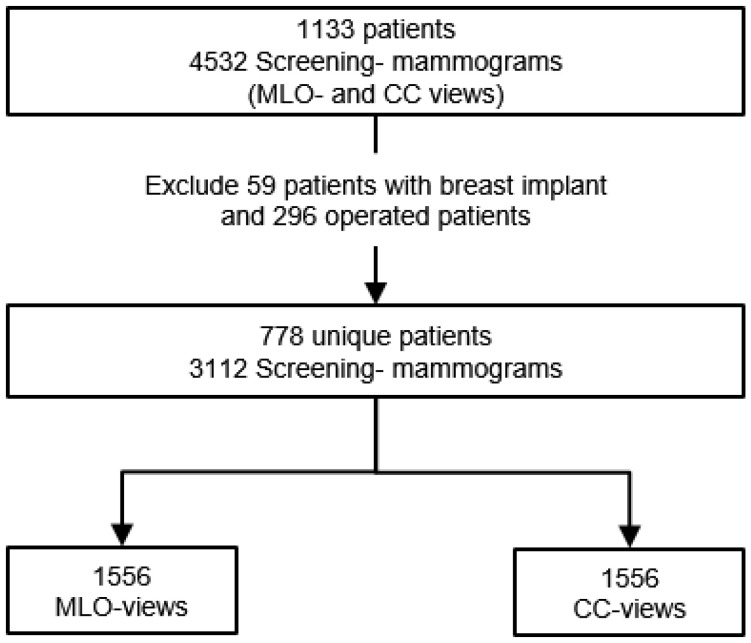
Flowchart of collected digital mammography studies. From 4532 screening mammograms a set of 3112 exams was selected after excluding mammography examination of operated patients and patients with breast implants.

Since only the INbreast dataset, with an insufficient number of mammograms, could be used, 1042 Screening mammography studies from 6 different radiology departments in Germany and abroad were additionally collected for the purpose of this study. All mammograms were full-field digital images of patients aged 19 to 89 years (mean age: 56.4) performed between April 2014 and May 2021. The collected images are in DICOM format and have a high resolution of at least 1770 × 2370 pixels. Mammography studies of operated patients (*n* = 296) and patients with breast implants (*n* = 59) were excluded (Figure 2). The remaining 778 screening mammography studies yielded a total of 1556 MLO and CC views for each breast and will be assessed according to the predefined breast positioning quality criteria.

**Figure 3:**
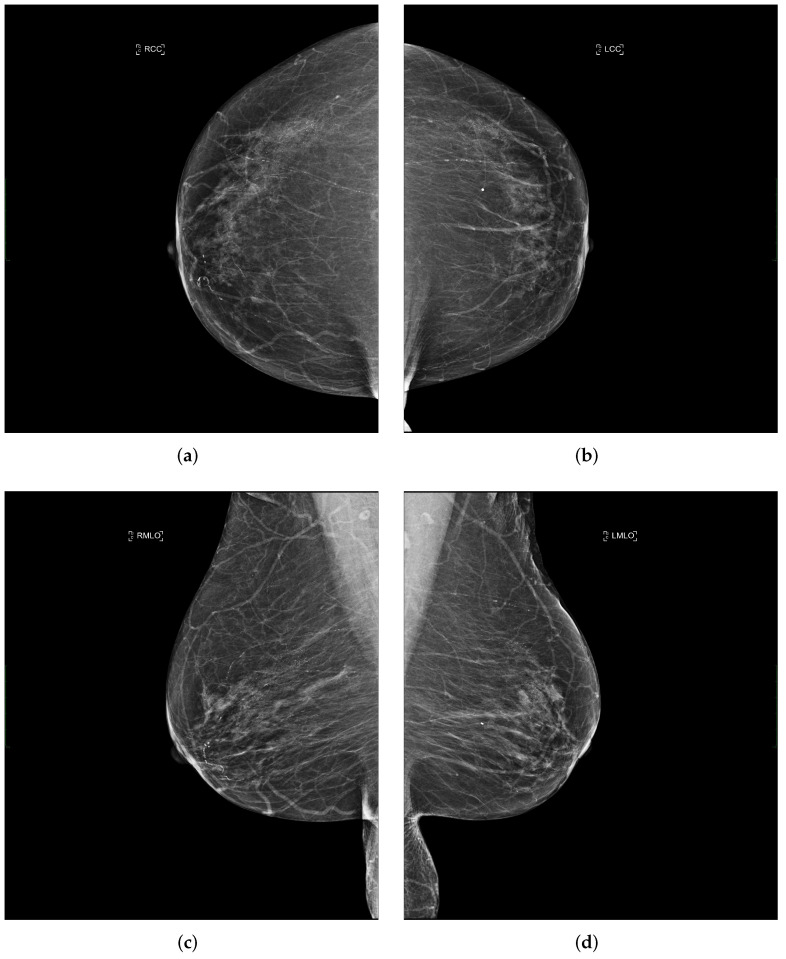
Example of a mammography study selected from the collected dataset showing the four standard views (**a**–**d**). (**a**) right cranio-caudal (R-CC); (**b**) left cranio-caudal (L-CC); (**c**) right mediolateral oblique (R-MLO); (**d**) left mediolateral oblique (L-MLO).

Each performed mammography exam includes four standard views: a CC and a MLO view for each, the right and left breast, as illustrated in Figure 3.

Clinical image quality criteria for assessing correct breast positioning in mammography screening were initially derived from the Mammography Quality Standards Act (MQSA) published by the American College of Radiology (ACR) [16] and effective mammography research by Bassett et al. [17,18]. In addition, several national and international quality standards have been published to ensure adequate breast positioning, for instance, the United Kingdom National Health Service Breast Screening Program in 2006 [19], the European Guidelines for Quality Assurance in Breast Cancer Screening and Diagnosis in 2006 [20], and BreastScreen Aotearoa, New Zealand, in 2016 [21].

### 2.1. Mediolateral-Oblique View

**Figure 4:**
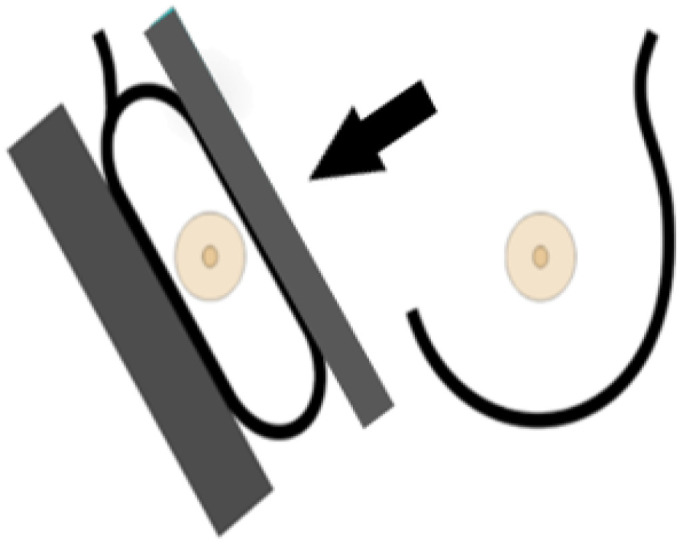
Mediolateral-oblique imaging technique [22].

**Figure 5:**
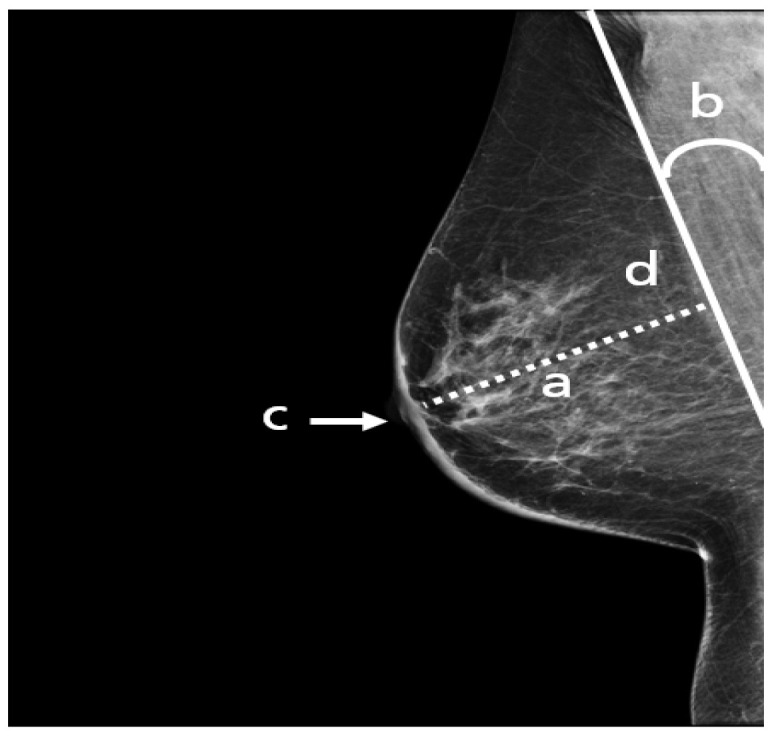
An adequate MLO view with (a) pectoralis to nipple level, (b) relaxed with an angle > 10°, (c) nipple in profile, and (d) visualized retroglandular fat.

A standard view taken from the medial upper end to the lateral lower end at an angle of approximately 45° (Figure 4). It represents the most important projection as it allows visualization of the upper outer quadrant with the greatest amount of breast tissue. In an adequate MLO view, as shown in Figure 5, the pectoralis muscle should be relaxed, imaged to the nipple level (a) and convex or straight, with an angle of a least 10° (b), the nipple must be in profile (c), and all the breast tissue and the retroglandular fat should be clearly shown (d), with no skin folds present.

### 2.2. Cranio-Caudal View

**Figure 6:**
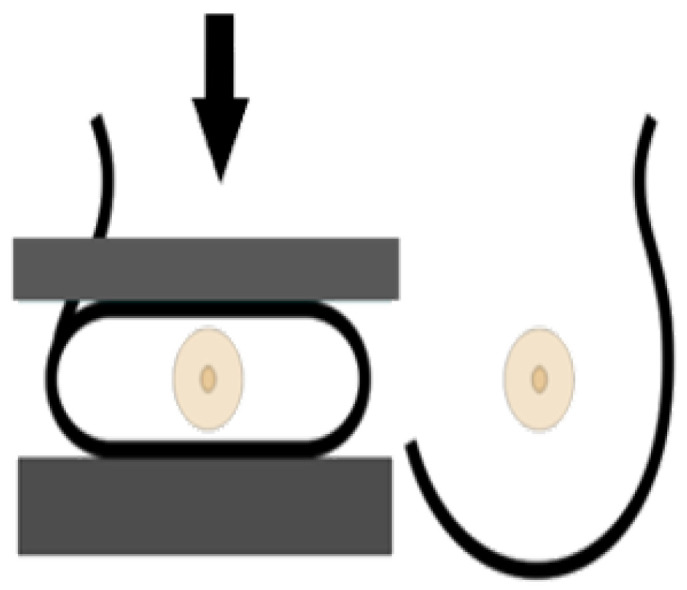
Cranio-caudal imaging technique [22].

A standard view taken from above in which X-rays propagate vertically downwards from the top of the chest (zero position of the X-ray tube), as shown in Figure 6. This projection provides a fully covered imaging of the glandular tissue in the inner breast quadrant area, allowing the radiologist to see “behind” tissue that might obscure a lesion on the MLO view.

**Figure 7:**
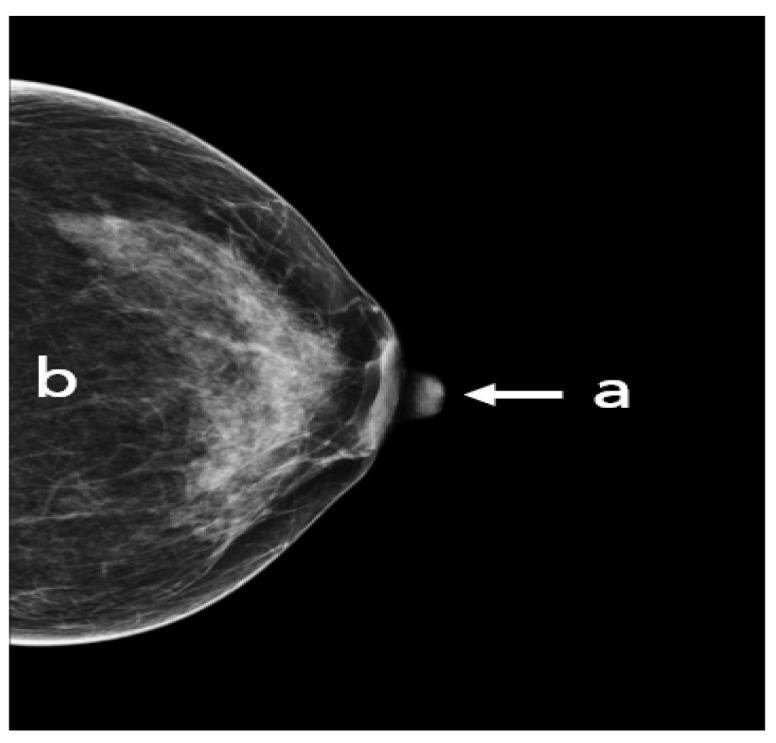
An adequate CC view with (a) nipple in profile and centered, (b) visualized retroglandular fat.

The following points have to be fulfilled in an adequate CC view (Figure 7): the nipple should be in profile, ideally centered (a) or pointing no more than 20° laterally or medially. All breast quadrants must be adequately visualized and the retroglandular fat (b), a fatty tissue closest to the chest wall and appearing as a dark stripe, must be present. The mammograms should not show skin fold obscuring diagnostically relevant parts of the glandular tissue.

### 2.3. Data Classification

The remaining 778 screening mammograms after exclusion of operated patients and patients with breast implant were classified according to the above quality criteria for breast positioning in both MLO and CC views. A separate dataset was created for each quality criterion, with two different classes of mammograms meeting or not meeting the respective criterion. Furthermore, this quality assessment was reviewed by a specialist radiologist with five years of experience in mammography imaging to ensure a reliable classification of the data.

**Figure 8:**
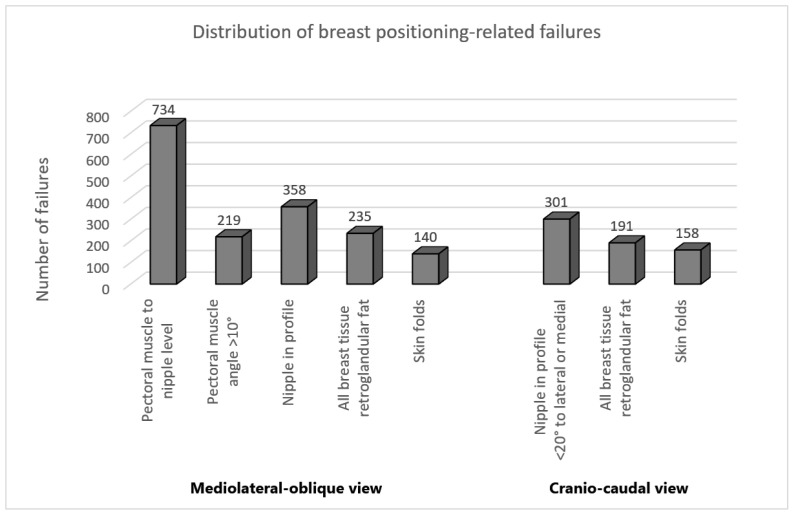
Distribution of the positioning-related failures of the assessed MLO and CC views.

The distribution of breast positioning related failures for both MLO and CC views is illustrated in Figure 8. Interestingly, analysis of our data confirms the results of previous studies [23,24] showing that the level of the pectoralis muscle was by far the most frequently missed criterion in MLO view following the visualization of the inframammary angle. This criterion was not fulfilled in 47% of the classified mammograms. In the CC view, the incorrect positioning of the nipple was the most common error with 19% of the evaluated mammograms.

The main goal of this work is to develop convolutional neural networks for an automated evaluation of breast positioning quality in mammography. To achieve this, it must be ensured that the implemented convolutional neuronal networks only consider the features related to image quality in terms of breast positioning learning without confusing these criteria with the ACR classification or with the detection of breast cancer. For this purpose, in addition to assessing breast positioning, all mammography studies were evaluated according to their ACR classification and divided into two groups: Mammograms with typical lesion types such as masses or microcalcifications and mammograms showing no abnormalities.

**Figure 9:**
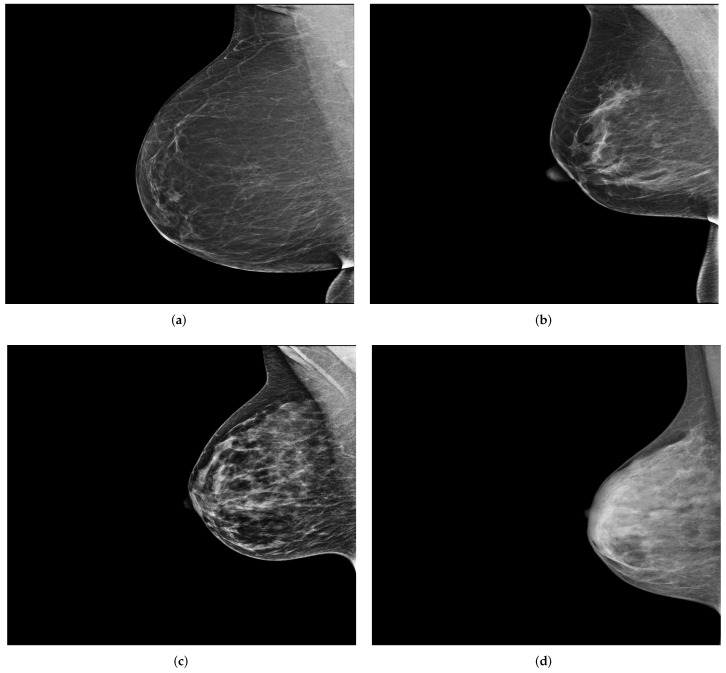
Example of the four breast density categories from the collected dataset. (**a**) ACR1: almost entirely fatty; (**b**) ACR2: scattered areas of dense fibroglandular tissue; (**c**) ACR3: heterogeneously dense; (**d**) ACR4: extremely dense.

The ACR classification of the American College of Radiology refers to the density of the breast according to the amount of fibroglandular tissue relative to fat in the mammogram. Increased glandular tissue also increases the risk of not detecting pathological findings and small features in the mammogram. Breast density classification is an essential part of breast cancer screening helping radiologists to interpret and report back mammogram findings. According to this classification, the density distributions are divided into four categories, as shown in Figure 9.

The classification results shown in both Table 1 for MLO views and Table 2 for CC views indicate that all ACR categories are present in each of the two predefined classes with good and poor breast positioning quality. Distribution of breast density is age dependent [25]. With advancing age, the amount of fatty tissue compared to glandular tissue usually increases. For this reason, it is to be expected that the majority of the collected mammograms belong to both categories ACR1 and ACR2, as the average age of the evaluated patients was 56.4 years.

**Figure 10:**
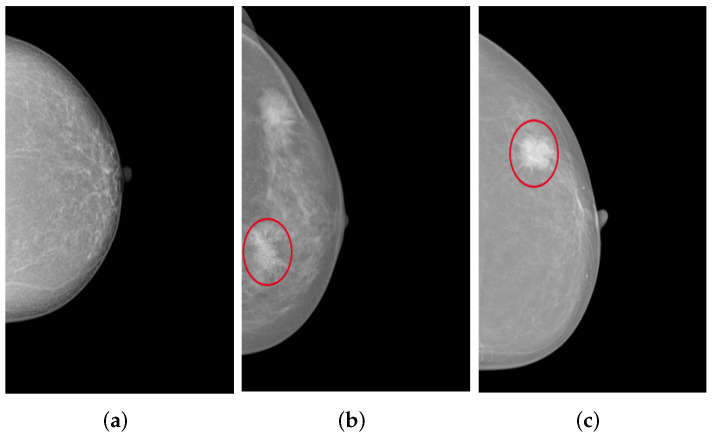
CC views of the left breast selected from the collected dataset: (**a**) shows no lesions, (**b**) shows fine and grouped microcalcifications in the inner lower quadrant of the breast (cf. red circle), and (**c**) illustrates a spiculated mass in the outer upper quadrant of the breast (cf. red circle).

The collected mammograms were additionally evaluated by a specialized radiologist regarding the presence of the two typical types of lesions in the breast tissue: Masses and microcalcifications, as shown in Figure 10. The classification indicates that 37.4% of MLO views with poor breast positioning quality and 33.4% of those with good breast positioning quality correspond to pathological patients. On CC views, 38.5% of poorly positioned mammograms and 28.8% of adequately positioned ones revealed either masses, microcalcifications, or both.

Having all ACR categories and finding types represented in both predefined positioning quality classes (good/poor) ensures that the model can learn the quality criteria related to breast positioning and thus achieve higher classification performance.

### 2.4. Data Preprocessing and Augmentation

**Figure 11:**
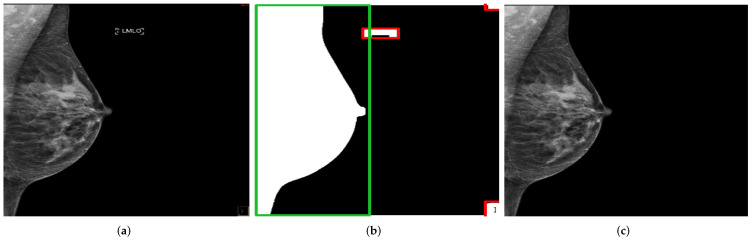
Recognition and removal of all irrelevant information. Green box with the detected chest area and red boxes with all irrelevant detected objects. (**a**) Original MLO view; (**b**) dilation image and objects recognition; (**c**) generated mammogram showing only the breast area.

For the training, necessary preprocessing steps were performed to consider, as far as possible, only the features related to breast positioning quality. This would allow a highly accurate and reliable model prediction. All irrelevant features on mammograms that may negatively influence the classification task must be removed. In most cases, these tend to be texts referring to the scanned breast side (right/left) and the standard mammography views (MLO/CC), which are located in different positions on the mammograms. For this purpose, using the OpenCV library, a method was implemented to automatically recognize and remove texts and then return a mammogram showing only the breast area. The procedure is illustrated in Figure 11.

The distribution of the collected mammograms regarding the breast positioning quality criteria Figure 8 reveals a problem of unbalanced data. Mammograms with inadequate breast positioning quality are of great interest for this study, however they represent the minority class for all standard criteria except the criterion for assessing the height of the pectoralis muscle in the MLO view.

Class imbalance has been demonstrated to have a significant negative impact on the classification performance of convolutional neural networks. It affects both convergence in the training process and the generalization of the CNN model on the test dataset [26]. As a result, learning and accurately predicting the minority class becomes much more difficult and, in worst cases, no longer possible.

**Figure 12:**
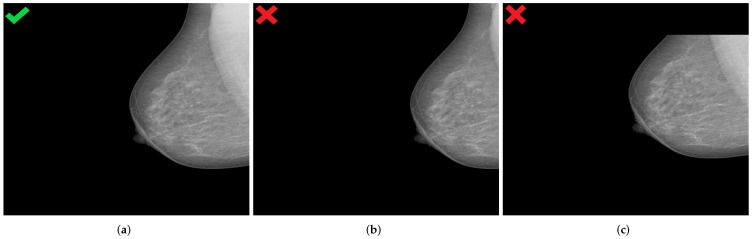
Generated mammograms with poor positioning quality by applying appropriate horizontal shift (**b**) and vertical shift (**c**) from an originally well-positioned mammogram (**a**). (**a**) Well-positioned mammogram (MLO view); (**b**) pectoralis muscle showing improper height and angle; (**c**) missing breast area in upper quadrant.

Image data augmentation is a technique used in deep learning algorithms to artificially increase the size and variability of a training dataset by creating modified versions of already existing images in the dataset. From the standard transformations, we applied random horizontal flips to introduce lateral (left or right) invariance. In addition, an automatic horizontal and vertical translation of the originally selected mammogram was used to generate new images with poor breast positioning quality even from an initially well-positioned mammogram (Figure 12).

**Figure 13:**
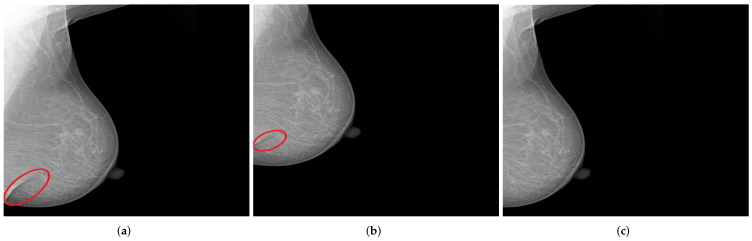
Examples of generated mammograms showing the significance of the selected shift value. (**a**) Original mammogram with a skin fold (cf. red circle); (**b**) created mammogram with a preserved skin fold (cf. red circle); (**c**) missing skin fold due to horizontal shift with an inappropriate value.

Shift values for both translation types must be carefully selected to ensure that the features relevant for model prediction are preserved, as illustrated in Figure 13.

**Figure 14:**
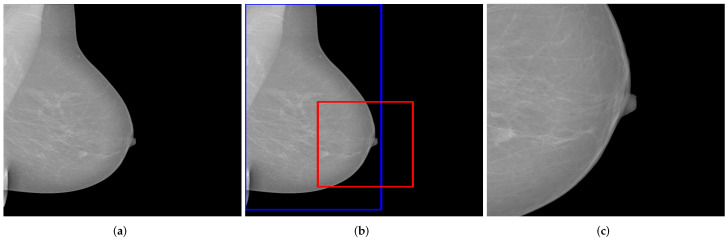
Example of a generated mammogram showing the nipple in a relatively enlarged area. The blue bounding box represents the identified breast area and the red box represents the cut-off area. (**a**) original mammogram; (**b**) identifying the breast and the cut-off-area; (**c**) resulting mammogram using the crop function.

The assessment of the nipple positioning is basically independent of the representation of other breast parts. For this reason, a crop function could be used in addition to the translation function in order to increase the number of mammograms with regard to this quality criterion, see Figure 14.

The implemented augmentation methods increase the numbers of mammograms in the minority classes so that they approximately match the numbers in the majority classes. Only for the criterion assessing the presence of skin folds in both MLO and CC views could the problem of class imbalance not be solved. Due to the small number of mammograms with pronounced skin folds, the evaluation of this quality criterion was excluded from the study.

### 2.5. Convolutional Neural Networks (CNN)

Convolutional Neural Network is one of the most impressive and commonly employed algorithms in the field of deep learning proposed by Yann LeCun et al. in 1998. This particular form of Artificial Neural Networks represents the mainly used model in medical image diagnosis and analysis. In fact, artificial intelligence for breast cancer detection in screening mammography [1,9,10,11] and computer-assisted detection and diagnosis (CAD) systems [27,28,29,30,31] have been successful thanks to CNN application. This substantiates the use of CNN in our study for automated assessment of breast positioning quality in screening mammography.

**Figure 15:**
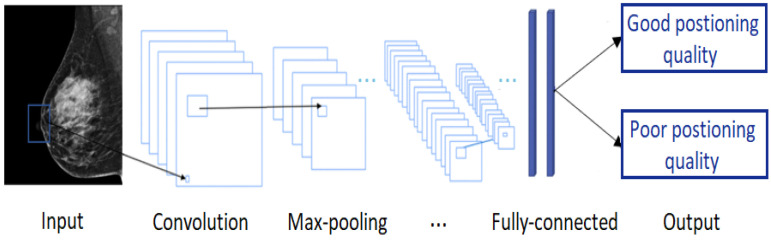
A typical convolutional neural network architecture.

CNNs are applied to explore patterns in an input image by convoluting over it, looking for the relevant features and assigning weights to different aspects of the image in order to differentiate it from the other ones in the training data. During the learning process, lines, corners, and edges can be recognized in the few front layers. More complex features can be detected as the network gets deeper. This property ensures that CNNs are very efficient in object detection and image classification [32]. Generally, the architecture of a CNN consists of three main layers, namely convolutional layers, pooling layers, and fully linked layers [33], as shown in Figure 15.

The convolutional layer is the first layer in CNN architecture where the majority of computation occurs. It is composed of several small-area filters, such as 3 × 3, 5 × 5, and 8 × 8 moving over the input image with a predefined stride to learn the recurrent patterns appearing in each area of the image. The pooling layer, also known as downsampling, is typically used at the output of each convolutional layer to reduce the dimensionality of the feature maps produced, decreasing the complexity of the computation while preserving the relevant features [34]. Another significant advantage of this layer is solving the invariance problem of feature maps due to a small change in the position of the recognized features by the convolutional layers [35]. The matrix resulting from both convolutional and pooling layers must first be flattened into a vector and then fed into the fully connected layer where the classification task is done. Here, all neurons in the previous and current layer are connected together [36].

## 3. Development of the CNN Models

With the exception of the criterion for assessing the presence of skin folds due to lack of data, a separate CNN model was developed for each predefined quality criterion in both MLO and CC views with a corresponding dataset. All mammograms are in JPEG-format, saved and resized to an identical selected dimension depending on the criterion to be assessed. We have assured that all sub-datasets (training, validation, and testing) include mammograms corresponding to the four different ACR categories and include both normal and pathological results. This ensures that the models focus on breast positioning quality during training. In addition, it can then be verified whether the breast density or the presence of typical lesion types in the breast tissue negatively affects the model predictions.

The mammograms were automatically labeled during the loading process. Each folder represents a class (good/poor) and contains the corresponding mammograms with regard to the respective quality criteria. Thus, the task is interpreted as a binary classification. Our architecture is based on the sequential model, which is appropriate for a plain stack of individual layers where each layer has exactly one input tensor and one output tensor [37]. The grayscale values (0–255) of all 8-bit mammograms in the datasets were normalized before being used as input for the network. For each implemented model, several training parameters such us the batch size, the epochs number and the learning rate were optimized. Furthermore, hyper-parameters including the filter size of the different convolutional layers and stride were varied during the different trials to find the optimal values, leading to the best possible classification results. As activation functions we used ReLUs, with the exception of the Output Layer. There, the Softmax activation was performed, which provides the probability of class membership. We used Adam as the optimization algorithm and Categorical Crossentropy as the loss function. In addition, dropout, L1 and L2 regularization methods were applied in order to prevent overfitting.

### 3.1. MLO: Pectoralis Muscle Angle

**Figure 16:**
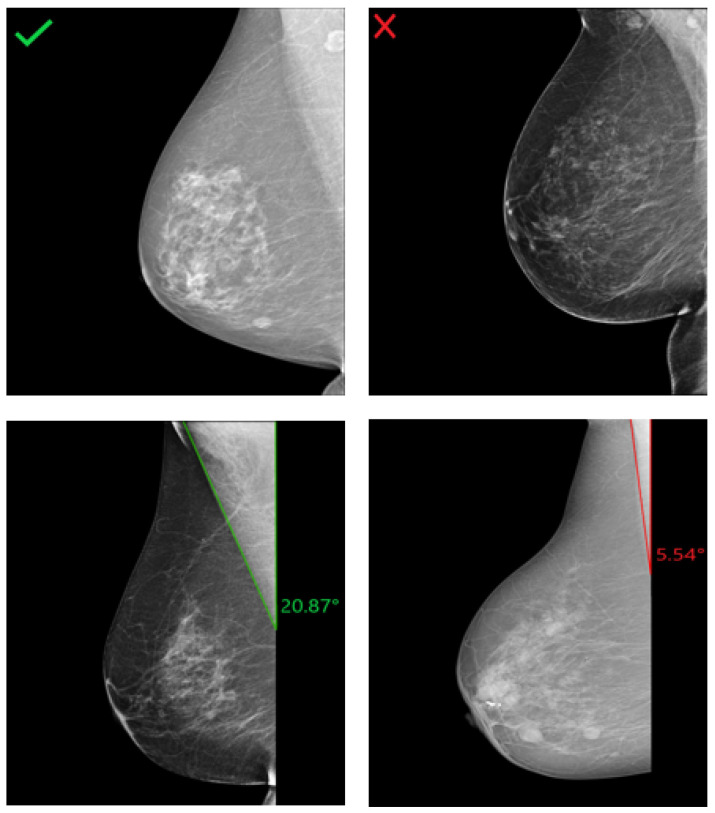
Assessment of the pectoralis muscle angle in four different R-MLO views. (**Left**): Mammograms with correct pectoralis muscle angle. (**Right**): Inadequate positioned mammograms due to convex pectoralis muscle (above) and angle <10° (below).

In an adequately positioned mammogram, the pectoralis muscle margin should be well visualized, triangular, or slightly concave with an angle >10°. These criteria are required to visualize the maximum amount of breast tissue on the clinical images. With decreasing angle, the risk of missing relevant breast tissue parts for mammographic cancer detection increases. Examples of R-MLO views with correct and inadequate pectoralis muscle angle are shown in Figure 16.

**Figure 17:**
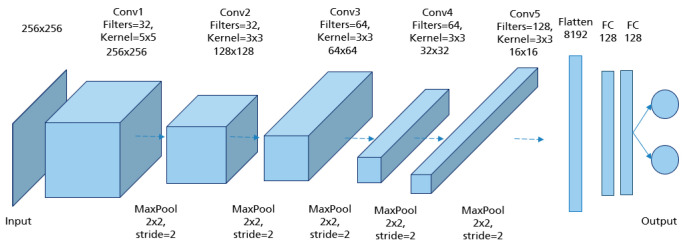
Model architecture of the implemented CNN for assessment of the pectoralis muscle angle with the best performance.

In order to obtain the model architecture with the appropriate hyperparameter combination, different CNN models were trained and evaluated. The model architecture in Figure 17 achieved the best prediction results with the highest accuracy of 94.3% reached in the testing set. The input of the network was MLO mammograms downscaled to 256 × 256 pixels. The image size was not randomly defined. It was selected based on the classification results of many performed trials. For each class (good/poor breast positioning quality), 1358 mammograms were used for training, 360 for validation, and 80 for testing. The number of epochs was set to 200 and the batch size to 32. Adam was used as the optimizer with a learning rate of 5 × 10${}^{-4}$. For the training, this network with its 5,045,058 learned parameters required about 6 h on the Nvidia GTX 1080 Ti graphics card.

To ensure that the implemented models work correctly, the gradient-weighed class activation mapping (Grad-CAM) approach was used, highlighting the particular region of the input image that are important for the classification decision [38]. This technique is a generalization of class activation mapping (CAM), which allows the visualization of the discriminative object parts detected by the CNN by replacing an existing fully connected layer with global average pooling (GAP) followed by a fully-connected softmax layer [39]. Unlike CAM, Grad-CAM does not require retraining of the network and works based on the feature maps of an input image and the specific gradient information flowing into the final convolutional layer of a CNN. The final color map or localization map is obtained by applying ReLU to the linear combination of maps to emphasize only the features that have a positive influence on the class of interest [38]. The linear combination is the summation of the pooled gradient using GAP multiplied by the obtained feature maps.

**Figure 18:**
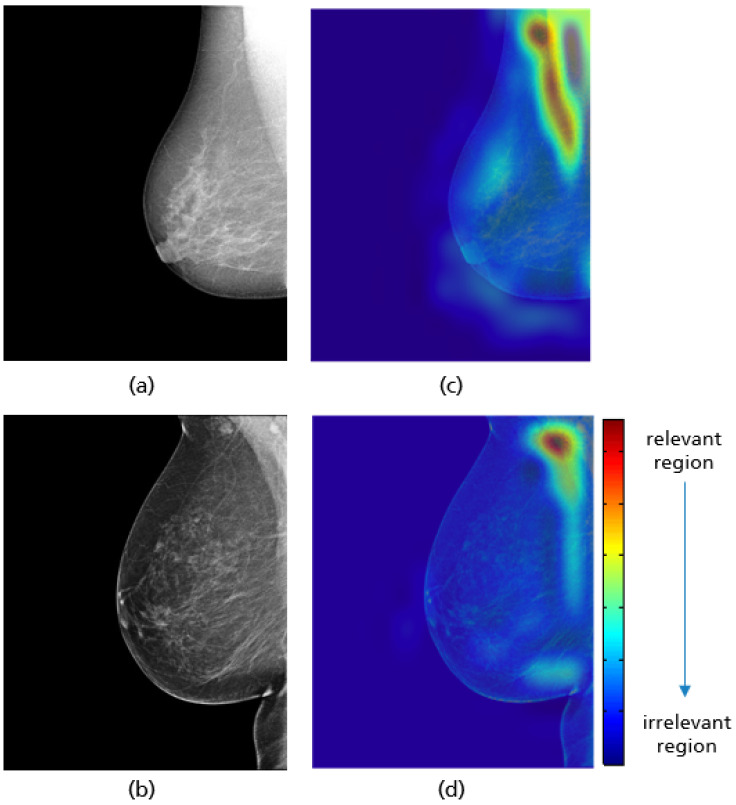
Original MLO views with correct (**a**), inadequate pectoralis muscle angle (**b**) and their Grad-CAM (**c**,**d**) highlighting the relevant region for the model prediction. Red indicates areas of high relevance. The color distribution extends to a blue area indicating low value.

**Figure 19:**
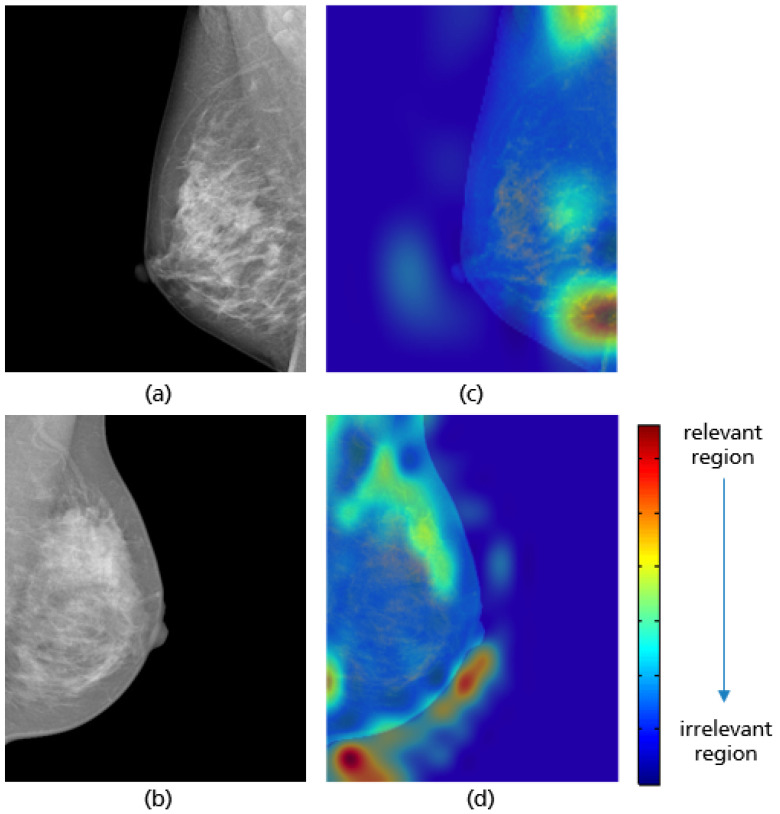
MLO view misclassified by the CNN model due to a low contrast (**a**) and the absence of retroglandular tissue (**b**), the corresponding CRAD-CAM visualizations (**c**,**d**).

For all mammograms in the test set, we first obtained class prediction from the implemented CNN model, then generated the Grad-CAM maps for each of the predicted classes (good vs. poor breast positioning quality). Grad-CAM visualizations of the model predictions in Figure 18 revealed that, as expected, the model had learned to look at the edge of the pectoralis muscle to evaluate its angle. This explains the high accuracy of the model (96%) and confirms the learning of the relevant features for the classification task. However, the model misclassified some mammograms, and from the Grad-CAM maps it was clearly identified that the reason for the failure was either the low contrast selected from the mammography technologist (Figure 19a) or the absence of the retroglandular tissue (Figure 19b).Both made the detection of the pectoralis muscle-border difficult or even impossible in some cases (cf. Figure 19c,d).

### 3.2. MLO: Pectoralis Muscle Level

**Figure 20:**
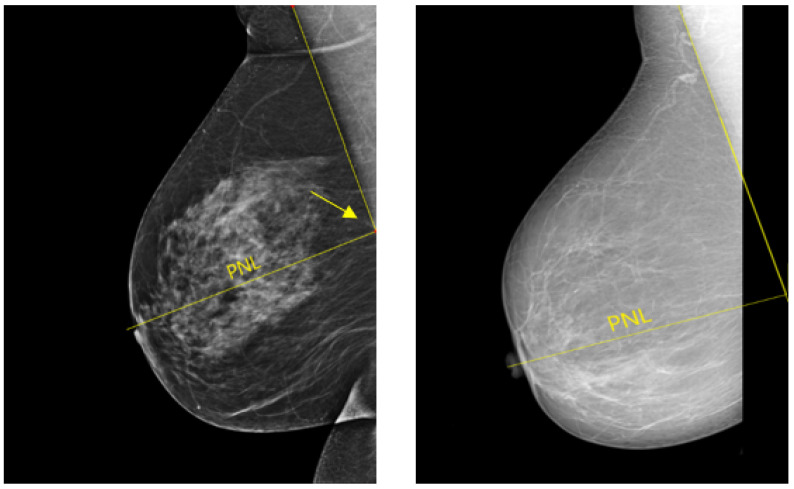
Example of adequately and inadequately positioned MLO views. (**Left**): a well-positioned MLO view in which the pectoralis muscle line and the posterior nipple line (PNL) intersect within the image area (cf. yellow arrow). (**Right**): an inadequately positioned MLO view where the pectoralis muscle line and the PNL do not cross within the image area.

In a well-positioned MLO view, the lower edge of the pectoralis muscle should be at the level of the posterior-nipple line (PNL) or below (see Figure 20). The PNL refers to a line drawn from the nipple posteriorly and perpendicularly towards the pectoralis muscle line [40]. This demonstrates that the mammogram includes adequate coverage of the posterior breast tissue. There is a risk of breast tissue exclusion, which is necessary for effective breast cancer detection, if the PNL does not intersect the pectoralis muscle line within the image area (Figure 20).

**Figure 21:**
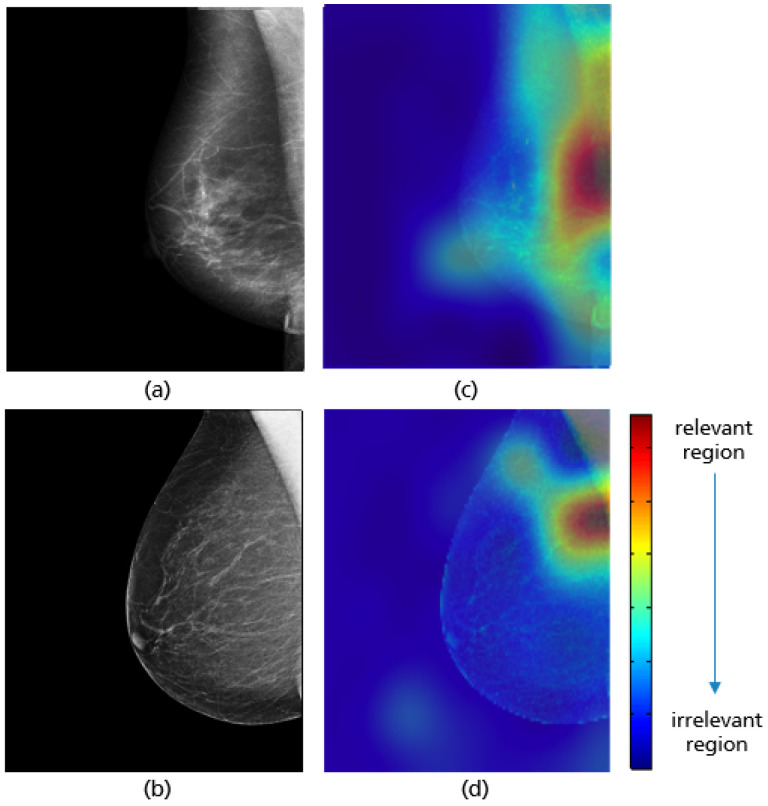
MLO views showing adequate (**a**) and poor positioning quality (**b**) with regard to the level of the pectoralis muscle and their Grad-CAM (**c**,**d**) highlighting the relevant region for the model prediction.

The same network architecture as in Figure 17 is used for the implemented model to evaluate the pectoralis muscle level. For each class (i.e., good vs. poor breast positioning quality), 1296 images are used for training, 180 for validation, and 80 for testing. Thereby, the input size of the images is set to 128 × 128 pixels. The highest model accuracy is about 96.8%, achieved by an 8-hour training over 150 epochs with a batch size of 32. As the optimizer, Adam was used, with a learning rate of 2 × 10${}^{-4}$. The Grad-CAM visualizations in Figure 21 prove that the CNN has learned the correct features for evaluating this quality criterion. Here, the lower edge of the pectoralis muscle corresponds to the region crucial for model prediction, which is highlighted in red for both adequately and inadequately positioned mammograms in Figure 21c,d. A possible source of the misclassified mammograms could be the low contrast, which makes the detection and recognition of the pectoralis muscle edge excessively difficult, as in the previous interpretation.

### 3.3. MLO: Nipple Position

**Figure 22:**
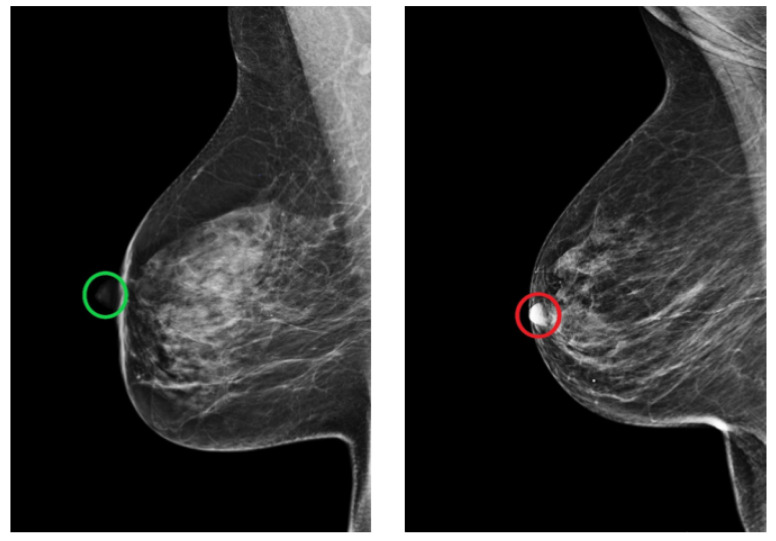
Example of adequately (green circle) and inadequately (red circle) positioned MLO views regarding the position of the nipple. (**Left**): in profile. (**Right**): projected onto the parenchyma.

The MLO view is also assessed by looking for the position of the nipple. In a well-positioned mammogram, the nipple should be in profile. Once it is projected onto the parenchyma, it may overlap a lesion and result in undetected malignancy. Figure 22 shows an example of adequately and inadequately positioned MLO views regarding the nipple position.

**Figure 23:**
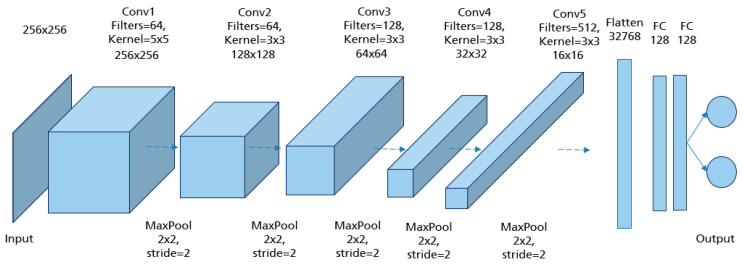
Model architecture of the implemented CNN for assessment of the nipple position with the best performance.

The best classification results, with an accuracy of 96.2% on the test data, were achieved by the model architecture shown in Figure 23. The collected augmented data is downscaled to 256 × 256 pixels and divided in such that for each class (i.e., good vs. poor breast positioning quality), 2428 images are used for training, 310 for validation, and 80 for testing. Training this network with its 5,045,058 parameters over 150 epochs with a batch size of 32 and a learning rate of 1 × 10${}^{-4}$ required approximately 10 h.

**Figure 24:**
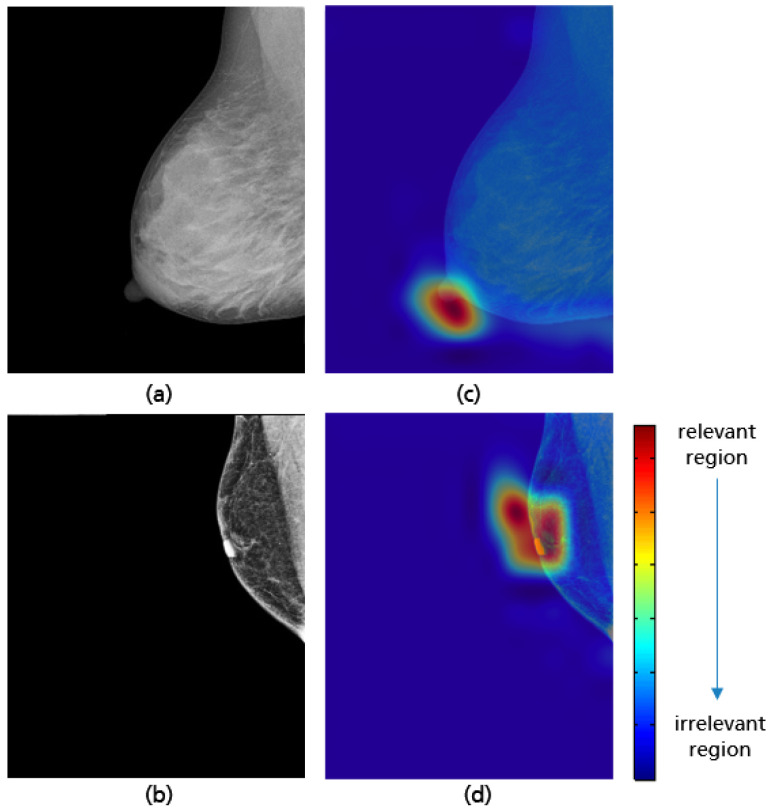
MLO views showing adequate (**a**), poor breast positioning quality (**b**) with regard to the nipple position and their Grad-CAM (**c**,**d**) highlighting the relevant region for the model prediction.

Mammograms in which the nipple was difficult to identify for anatomical reasons or due to a poor brightness ratio to the background were excluded from the study. This contributed in minimizing the false positive prediction and allowed the CNN to learn the relevant features during the training process. Grad-CAM maps of both adequate and inadequate positioned MLO views shown in Figure 24 demonstrate that the trained model only looks at the nipple position in the mammogram to evaluate the fulfillment of the corresponding quality criterion.

### 3.4. MLO: Coverage of All Relevant Breast Tissue

**Figure 25:**
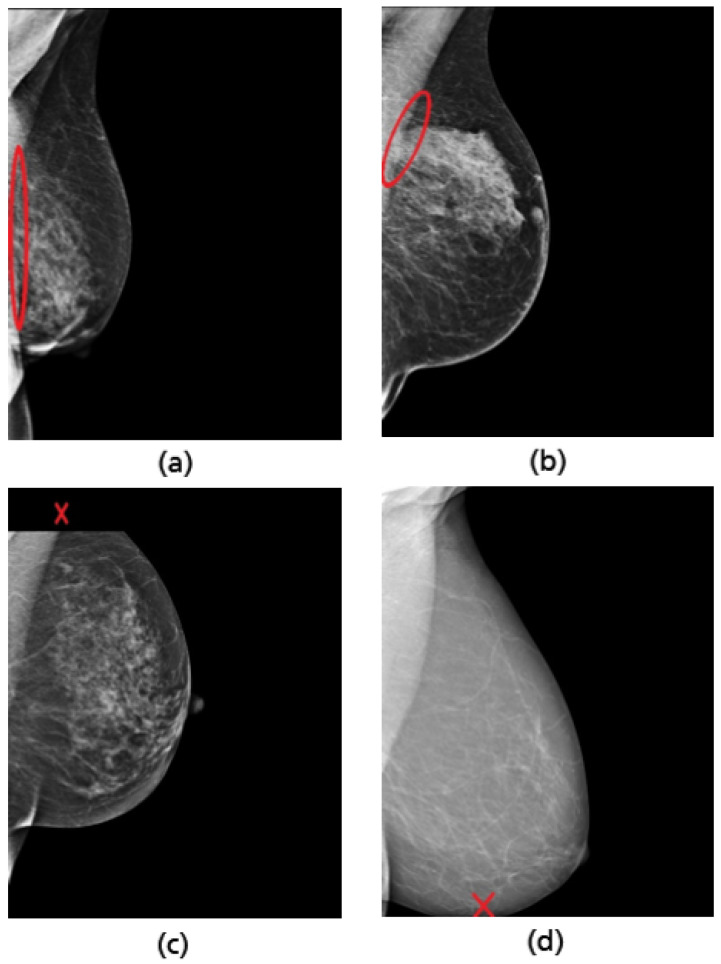
Examples of inadequately positioned MLO views. Retromammary tissue not fully visualized (**a**,**b**). Missing part of the axillary tail (**c**). Missing part of the lower breast quadrant (**d**).

The visibility of the axillary tail, retroglandular fat tissue, and the coverage of the entire breast tissue is the last criterion for assessing the positioning quality of an MLO view evaluated in this study. The L-MLO views in Figure 25 represent the different possible deficiencies with respect to this criterion.

To evaluate this quality criterion, an implemented CNN with the same model architecture as in Figure 23 and different hyperparameter values achieved the best classification results. For each class (i.e., good vs. poor positioning quality), 2294 mammograms were used for training, 180 for validation, and 80 for testing. The input data were downscaled to 128 × 128 pixels. Training the network over 200 epochs with a batch size of 32 and a learning rate of 1 × 10${}^{-4}$ took approximately 12 h. The implemented CNN model achieved an accuracy of 94.4% on the test data.

**Figure 26:**
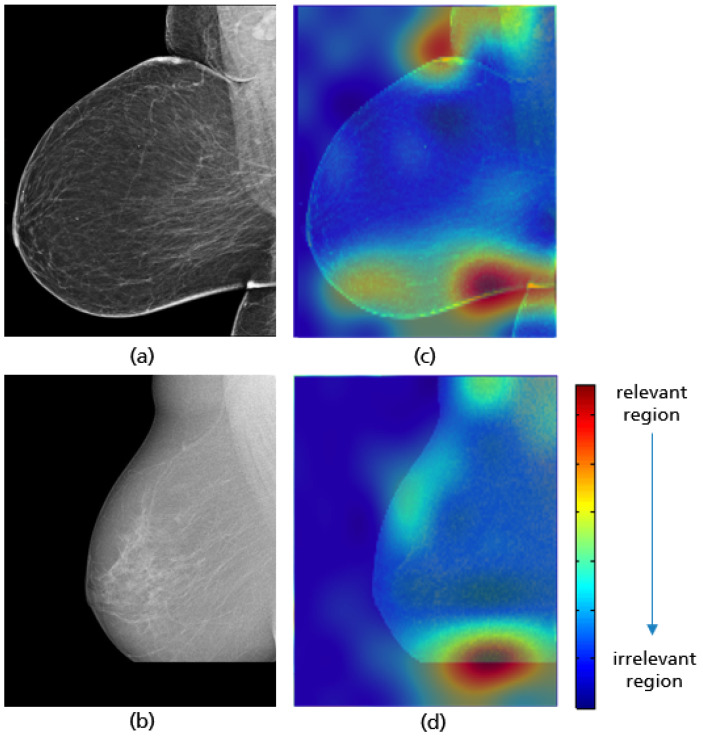
MLO views showing a well-positioned mammogram (**a**), an inadequately positioned mammogram with a missing breast part in the outer lower quadrant (**b**), and their Grad-CAM (**c**,**d**) highlighting the relevant region for the model prediction.

**Figure 27:**
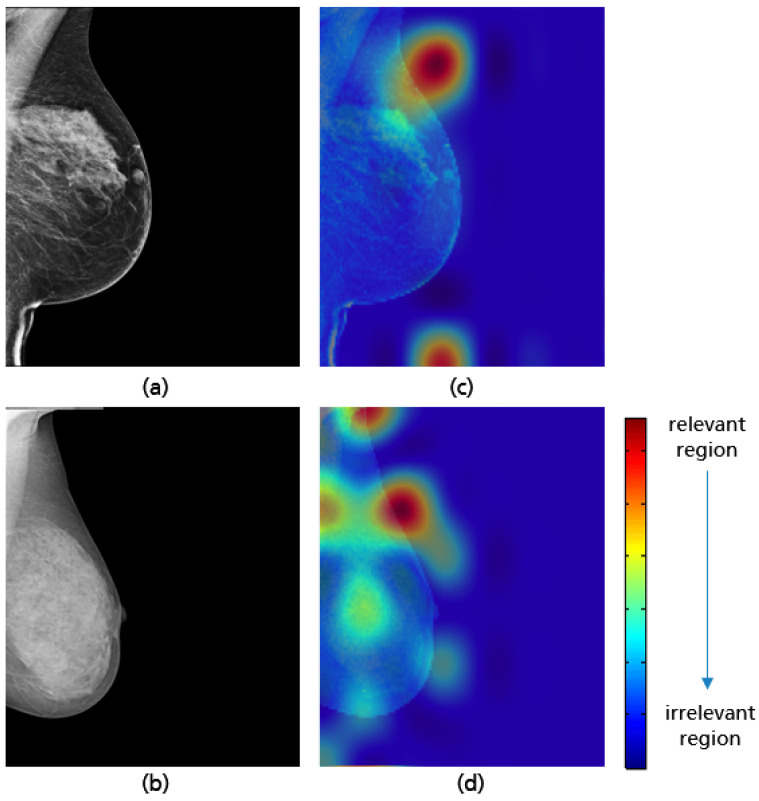
Examples of MLO views misclassified by the model with a not fully visualized retroglandular fat tissue (**a**,**b**), and their Grad-CAM (**c**,**d**) highlighting the relevant region for the model prediction.

Grad-CAM visualizations in Figure 26 reveal that the model considers both the upper and lower breast regions when classifying MLO views. In cases of incomplete imaging breast as in the example (b) in Figure 26 the CNN can reliably identify the position of the missing breast part. This demonstrates that the breast boundary is considered to be the relevant feature for the model’s prediction. However, the area of the retroglandular fat tissue remains unconsidered in most prediction cases (see Figure 27). This leads to misclassification when a not fully visualized retroglandular fat tissue remains the only deficiency present. The small amount of available data showing this failure could be the reason why this relevant feature was not adequately learned during the training process.

### 3.5. CC: Nipple Position

**Figure 28:**
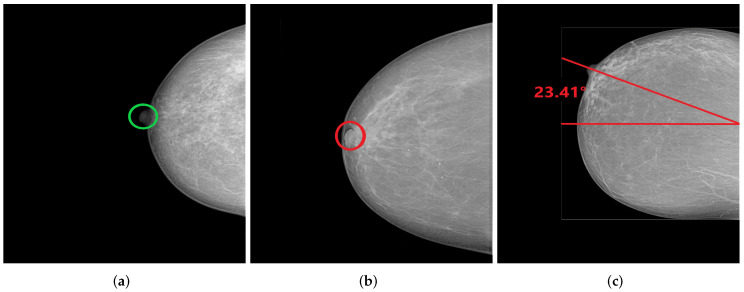
CC views with well-positioned nipple (**a**) and inadequately positioned nipple (**b**,**c**). (**a**) Nipple in profile; (**b**) Nipple on retromammary tissue; (**c**) Nipple deviated by 23.41° laterally.

In a cranio-caudal view, the nipple should be in profile as shown in Figure 28a to assure the visibility of the glandular tissue necessary for cancer diagnosis. The projection of the nipple onto the retromammary tissue may result in the masking of a lesion, leading to undetected breast cancer (see Figure 28b). Another positioning failure can also be the deviation of the nipple from the center by more than 20° medially or laterally and leads to an incomplete imaging of relevant lateral breast parts (see Figure 28c). This deficiency in breast positioning quality occurs in only 4% of all collected CC views. For this reason, the assessment of the nipple position in this study is limited to checking whether it is in profile or projected on the retromammary tissue.

The assessment of the nipple position is independent of all other breast parts. For this reason, the simultaneous use of both CC and MLO views during training and validation process is particularly useful, as more data improves the learning of relevant features and leads to a significant increase in the model performance. We used the same model architecture implemented for assessing the nipple positioning in MLO views in Figure 23. For each class (i.e., good vs. poor breast positioning quality), 3958 mammograms were used for training, 490 for validation and 80 for testing, downscaled to 256 × 256 pixels. Training this network over 150 epochs with a batch size of 32 and a learning rate of 1 × 10${}^{-4}$ took around 10 h.

**Figure 29:**
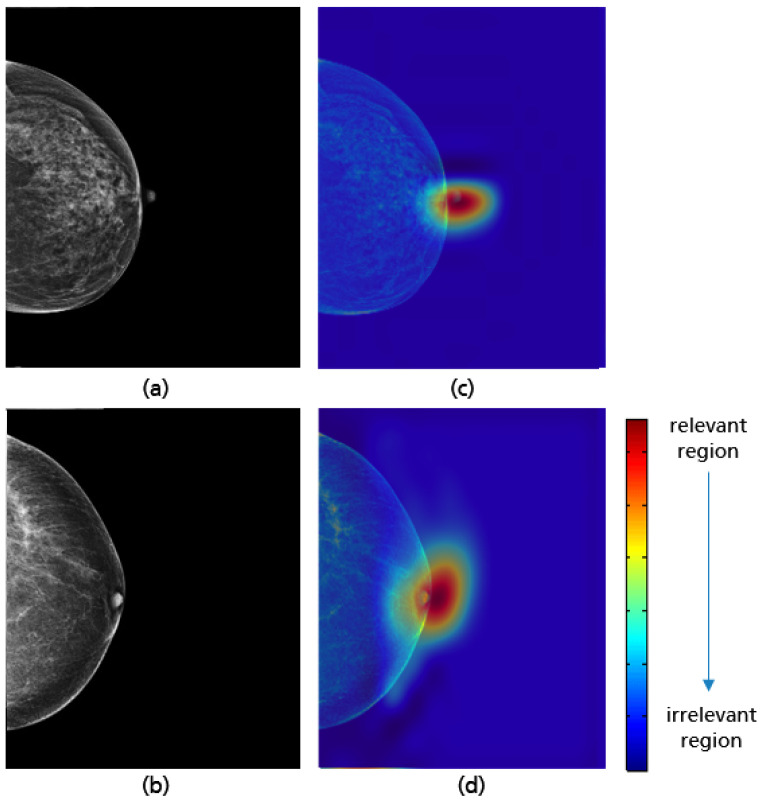
CC views with adequate (**a**), poor breast positioning quality (**b**) with regard to the nipple position and their Grad-CAM (**c**,**d**) highlighting the relevant region for the model prediction.

The model achieved a high accuracy of 98.2% using the test set. Based on the Grad-CAM visualizations in Figure 29, it is proven that the CNN is able to correctly identify the position of the nipple regardless of the breast size, which varies from patient to patient. When predicting the class of each CC view, only the position of the nipple on the mammogram was considered. This ensures that the model has learned the relevant features for this classification task and works as expected.

### 3.6. CC: Coverage of All Relevant Breast Tissue

**Figure 30:**
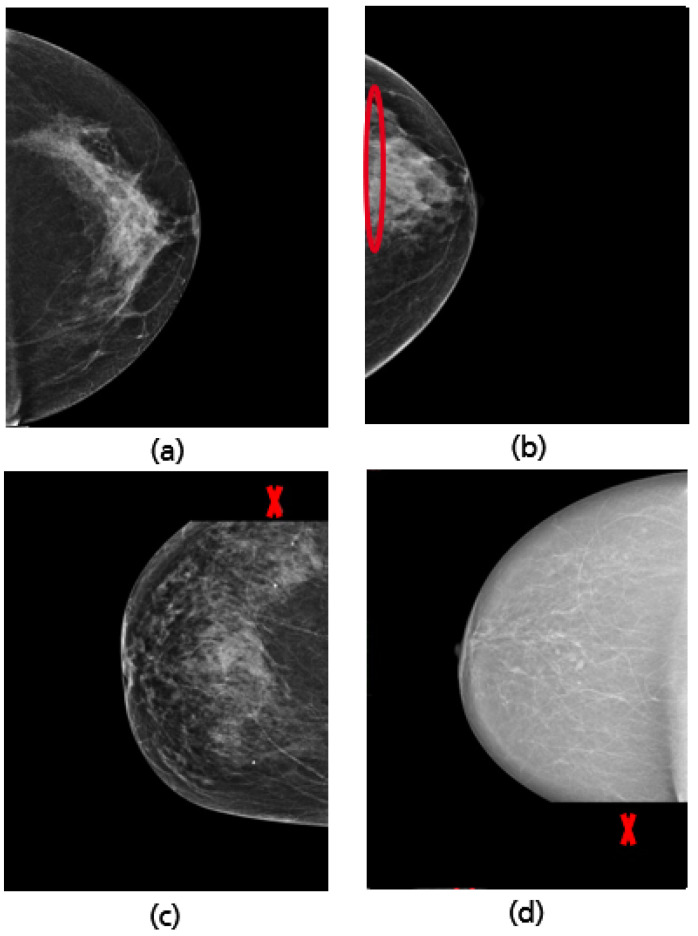
Examples of well-positioned (**a**) and inadequately positioned CC views: retromammary tissue not fully visualized (**b**), missing part of the axillary tail (**c**), and missing part in the lower breast quadrant (**d**).

The last positioning criterion assessed in this study is the imaging of the entire breast tissue on a CC view (see Figure 28b). It should be ensured that the maximum amount of mammary tissue is visible and no breast parts in the axillary tail, lower breast quadrant, and in the retromammary space are missed. The possible positioning failures are illustrated in Figure 30a–d.

**Figure 31:**
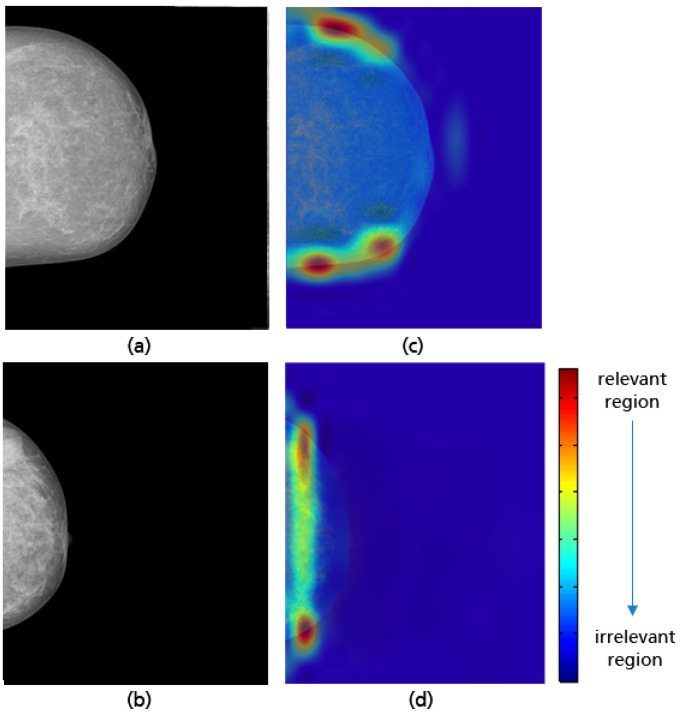
Examples of a well-positioned (**a**), an inadequately positioned CC view due to the visualized retrommamary space (**b**) and their Grad-CAM (**c**,**d**) highlighting the relevant region for the model prediction.

The best implemented CNN with the highest achieved accuracy of 97% has the same model architecture as in Figure 23. Per class (i.e., good vs. poor breast positioning quality), 1766 CC views are used for training, 180 for validation, and 80 for testing. All input data is downscaled to 256 × 256 pixels. Training the network over 200 epochs with a batch size of 32 and a learning rate of 8 required around 8 h. The GRAD-CAM visualizations demonstrate that the implemented model considers as expected the upper and lower breast edges to identify CC views with a complete imaging of all breast parts (see Figure 31a,c). This model has not only learned well the shape of a complete breast image (curved edge), but also correctly classified mammograms where the deficiency corresponds only to the retromammary space. The GRAD-CAM visualization example in Figure 31b,d illustrates that in such cases the CNN focused on the area of the retromammary tissue.

**Figure 32:**
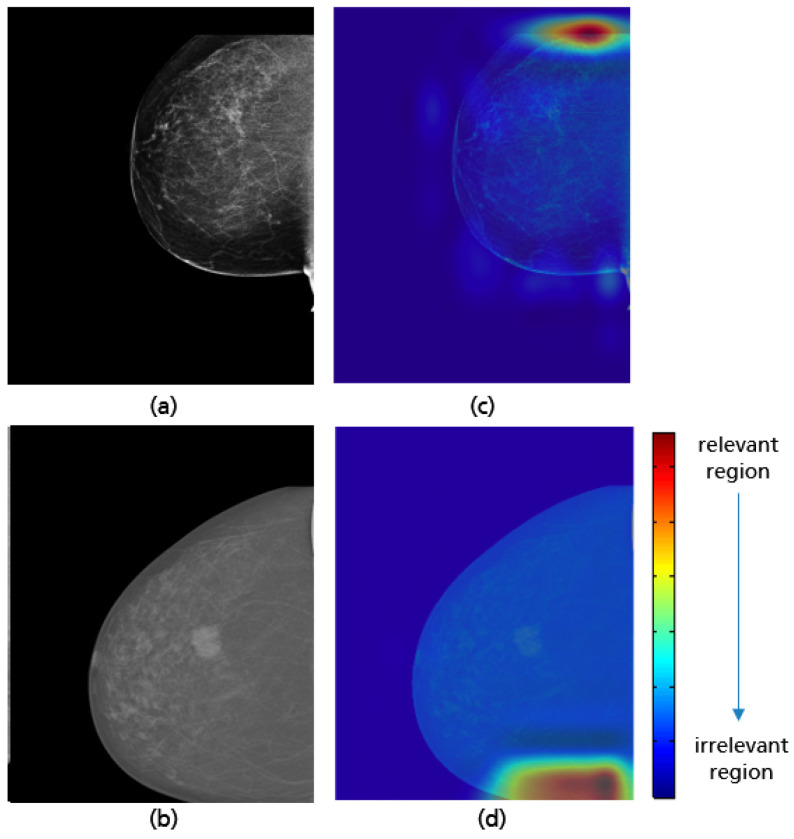
Examples of CC views with missing breast parts in the axillary tail (**a**) and in the lower quadrant (**b**), and their Grad-CAM visualizations showing the identification of the parts not covered during the model predictions (**c**,**d**).

**Figure 33:**
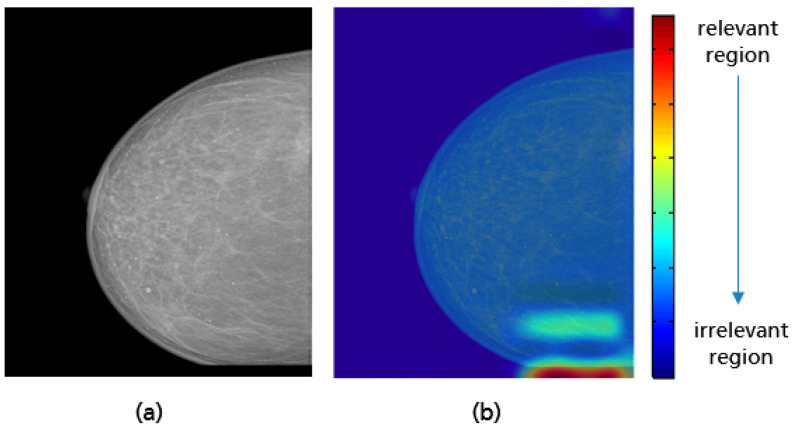
Example of CC view misclassified by the model with a small missing breast part (**a**) in the lower quadrant, and the Grad-CAM visualization (**b**).

When the axillary tail or the lower breast quadrant are not completely covered, as shown in Figure 32, the CNN is able to correctly identify the missing breast part and then make a reliable prediction. A review of the CC views misclassified by the model shows that the CNN has difficulty detecting the inadequately positioned mammogram when the missing breast part on the lower quadrant is too small (see Figure 33). This model limitation can be improved by using a large training dataset and thus the CNN can achieve better classification results.

## 4. Overall Systems and Results

**Figure 34:**
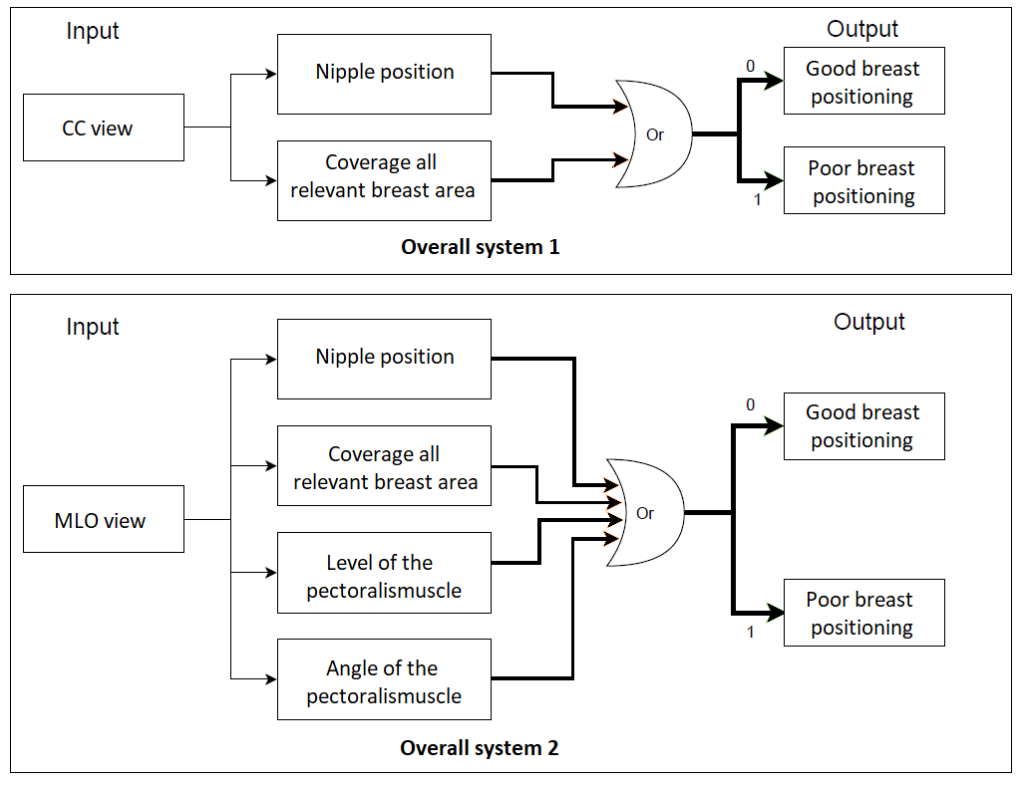
The overall process flow for the assessment of breast positioning quality in screening mammography. Overall system 1: Assessment of CC views, overall system 2: Assessment of MLO views. 0 refers to fulfilled quality criterion and 1 refers to non-fulfilled quality criterion.

For each standard view (CC and MLO), an overall system is built with the interconnection of all individual implemented classifiers (CNNs). The selected mammogram is entered as the input for each individual classifier within an overall system, which makes its own classification decision regarding the quality criterion to be considered. Subsequently, a final system prediction is made. The CC or MLO view is classified as a mammogram with poor breast positioning quality if at least one of the predefined quality criteria is not met. The entire process flow is clearly illustrated in Figure 34.

**Figure 35:**
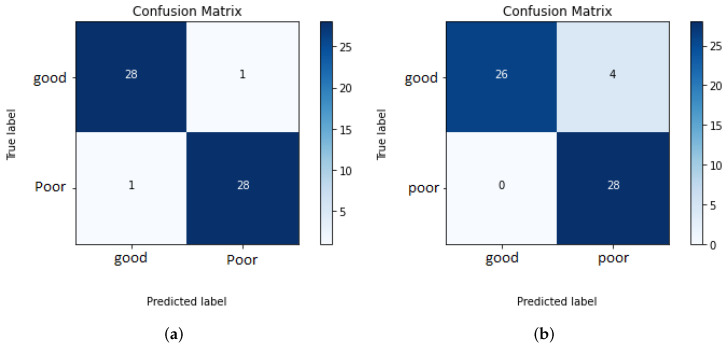
Confusion matrices of overall systems 1 and 2. (**a**) Assessment of the breast positioning quality by CC views. (**b**) Assessment of the breast positioning quality by MLO views.

To evaluate the built systems, a balanced test set of 58 mammograms for each view are used. The overall systems for assessing the breast positioning quality of CC and MLO views achieved an F1 score of 96.5% and 93.3%, respectively. The higher accuracy for the CC view can be explained by the fact that the overall system evaluates only two quality criteria and the individual CNNs showed so far better performance compared to those implemented for the assessment of the MLO view. The confusion matrices in Figure 35a,b provide the number of correctly classified (diagonal elements) and misclassified mammograms by the overall systems.

All MLO views showing poor breast positioning quality were correctly classified by the overall system. However, this does not necessarily mean that all individual classifiers made reliable predictions (see Figure 35b). The reason why only false-negative predictions occur by assessing MLO views is that most of the used test data do not fulfill more than one quality criteria at the same time. Therefore, it is sufficient to detect one of the presented deficiencies to correctly classify the input image as a mammogram with inadequate positioning quality. The evaluation of the system performance will be more effective and reliable by using a large test set consisting of more well-positioned mammograms as well as mammograms where only one criterion is not met.

## 5. Implementation as a Software Module for Clinical Decision Support

**Figure 36:**
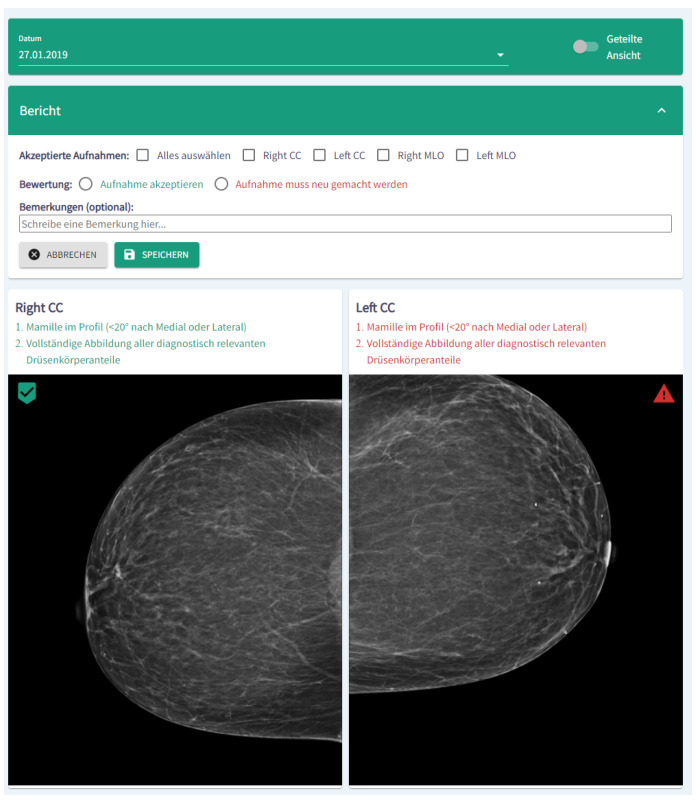
Screenshot (cutout) of the module visualization. In the upper part of the module, images can be accepted or rejected, whereas in the lower part the results of the algorithms are shown.

In order to provide exemplary results in a target group oriented way, the algorithms were integrated in a software module, which is embedded in a dashboard architecture. Figure 36 shows a cutout of the module visualization in German. In the upper part of the module, images can be accepted or rejected. Moreover, a short report and comments can be added. In the lower part, the results of the algorithms are shown. Please note: Figure 36 depicts only a cutout of the complete dashboard and the module visualization.

For implementation we used a NodeJS Express webserver and a single-page React Application. The web server manages the images, the neural network results, and stores the reports. Further preparations have been made so that the module can interact optimally with the Fast Healthcare Interoperability Resources (FHIR) [41,42] in the near future.

## 6. Discussion

In this contribution, we propose an approach for an automatic assessment of breast positioning quality in screening mammography using Convolutional Neural Networks. By guiding radiology technologists through identifying inadequately positioned mammograms in real time, we can reduce the number of returning patients with initially inadequate positioned image quality, improve the efficiency of cancer detection in screening mammography, and avoid additional radiation exposure to patients as well as extra hospital costs. The first challenge in our study was the collection and labeling of data, which is particularly difficult in the medical field. Thanks to the support of several radiological centers in Germany and abroad, a sufficient amount of data was obtained. Furthermore, the collected mammograms were pre-processed to remove all irrelevant image components that could lead to model misclassifications. The labeling of CC and MLO views regarding the positioning quality criteria derived from the Mammography Quality Standards Act (MQSA) was reviewed by a radiologist with five years of experience

In this work we trained a separate CNN classifier for the different quality criteria with a specific dataset. The choice of a deep convolutional neural network as the machine learning method to evaluate breast positioning quality was based on studies demonstrating that CNNs are the dominant approach, achieving the best accuracy on various medical image classification tasks [43]. In order to make our CNN models more understandable and to verify that they focused on the appropriate patterns in the screening mammogram to evaluate each specific breast positioning criterion, we used Grad-CAM, a class-specific gradient approach highlighting the relevant features for the model predictions.

All implemented models performed well, learned the relevant features, and achieved higher accuracy on the testing datasets (see Table 3 for more details). Different augmentation methods and optimization techniques were used to overcome the problem of imbalanced data and prevent overfitting of the models due to the limited dataset size, which lead to a better performance in classification. By combining the results of the separately implemented classifiers, we can predict whether the breast is well positioned or not with an accuracy of 96.5% for CC and 93.3% for MLO views through the overall built systems. Our approach offers promising results compared to the previous study [12], proposing to combine techniques from deep learning and feature-based machine learning algorithms to recognize inadequately positioned mammograms. Furthermore, the proposed models provided these classification results with minimal computational time and power, as we used an NVIDIA GTX 1080 graphics card in our study and the maximum time to train one network was about 12 h (the exact training times and learning parameters for each model can be found in the previous sections).

## 7. Conclusions

The results of our study prove that Convolutional Neural Networks have highly competitive performance and achieve accurate classification of screening mammograms in terms of breast positioning quality. In addition, the model prediction of each quality criterion could be provided to the radiology technologist in a structured report with a real-time feedback and precise information about presented positioning failures. This way, the breast could be repositioned correctly during the patient visit, ensuring the visualization of the maximum amount of mammary tissue.

Nevertheless, the study at hand has limitations that are worth acknowledging. The criterion for assessing the presence of skin fold in both CC and MLO views was not evaluated due to a small number of mammograms showing this deficiency. Moreover, mammograms in which the nipple was difficult to identify for anatomical reasons or due to a poor brightness ratio to the background were excluded from the study. As more appropriate datasets are obtained, it would be reasonable to consider these cases in further works. Consequently, a full assessment of the breast positioning in screening mammography will be performed by the developed overall systems. We can also enhance the performance of the proposed CNNs by collecting more inadequately positioned mammograms and including them in re-training our models. In conclusion, the achieved classification accuracy can be further improved by trying other deeper model architectures, combinations of hyperparameters, and by expanding the available training datasets as well as computational resources, although collecting medical image datasets is still a challenge since professional expertise is required for labeling.

## Figures and Tables

**Table 1:** Classification results of the collected 1556 MLO views according to ACR categories and quality of breast positioning.

ACR Category	Breast Positioning Quality	
	Good	Poor
ACR1	241	213
ACR2	387	291
ACR3	113	195
ACR4	78	38

**Table 2:** Classification results of the collected 1556 CC views according to ACR categories and quality of breast positioning.

ACR Category	Breast Positioning Quality	
	Good	Poor
ACR1	312	142
ACR2	472	206
ACR3	206	102
ACR4	84	32

**Table 3:** Classification results of all implemented models for the assessment of breast positioning quality in both MLO and CC views.

MLO View		
**Positioning Quality Criteria**	**Accuracy**	**F1-Score**
MLO: Nipple position	96.2%	96.3%
MLO: Coverage of all relevant breast area	94.4%	94.3%
MLO: Pectoralismuscle Angle	94.3%	94.2%
MLO: Pectoralismuscle Level	96.8%	96.9%
**CC View**		
**Positioning Quality Criteria**	**Accuracy**	**F1-Score**
CC: Nipple position	98.2%	98.3%
CC: Coverage of all relevant breast area	97.0%	97.0%

## Data Availability

The data presented in this study are available on request from the corresponding author. The data are not publicly available due to internal restrictions.
